# Presentation of Ascariasis as a Cause of Acute Intestinal Obstruction

**DOI:** 10.7759/cureus.62881

**Published:** 2024-06-22

**Authors:** Vasu Saini, Anjani Mahesh Kumar Cherukuri, Bhumika Bheemavarapu, Gnaneswar Pendurthi, Aniket Khamar

**Affiliations:** 1 Pediatrics, Shri Guru Ram Rai Institute of Medical and Health Science, Dehradun, IND; 2 Medicine, Guntur Medical College, Guntur, IND; 3 Medicine, Jawaharlal Institute of Postgraduate Medical Education and Research, Pondicherry, IND; 4 Medicine, Kempegowda Institute of Medical Sciences, Bangalore, IND

**Keywords:** x-ray images, ascaris lumbricoides, intestinal parasitosis, acute abdomen, ascariasis

## Abstract

Ascariasis infection is prevalent in tropical and subtropical locations worldwide. The occurrence of ascariasis is associated with poverty, inadequate hygiene, and inadequate fecal sanitation. This helminth often resides innocuously in the small intestine, but it may sometimes lead to intestinal blockage or perforation, resulting in peritonitis, a condition frequently seen in children. Ascariasis may also migrate via the ampulla of Vater, leading to the development of cholangitis, pancreatitis, cholecystitis, and, in rare cases, hepatic abscesses. Occasionally, an Ascaris-induced hepatic abscess may manifest as an acute abdomen, resembling an acute pyogenic abscess. We report the case of a four-and-a-half-year-old male child from Khedi Sikarpur, Haridwar, India, who was admitted to the pediatric department. The patient presented with acute colicky abdominal pain localized to the abdomen, accompanied by vomiting and constipation for three days. Additionally, the patient experienced abdominal distension for one day. There was no history of bleeding manifestations or decreased urine output.

## Introduction

*Ascaris lumbricoides* is a commonly identified helminthic parasite that frequently infects humans. Ascariasis, the condition caused by this parasite, is still prevalent worldwide, with about 1.2 billion infections resulting in over 60,000 deaths annually [1]. The prevalence of this condition is particularly high in the Middle East and South America, particularly in underdeveloped nations where inadequate sanitation, which is the primary risk factor for infection, is widespread [2]. Infection with *A. lumbricoides *is prevalent across all age groups, with a higher incidence seen among preschool children. While ascariasis infections often do not show symptoms, the infection may result in malnutrition in children and is responsible for between 3,000 to 60,000 fatalities annually, mostly due to intestinal blockage [3,4]. This infection is common among individuals residing in tropical and subtropical regions globally. The occurrence of ascariasis is correlated with poverty, inadequate hygiene, and deficient fecal sanitation.

Infection with Ascaris begins when embryonated eggs are ingested. These eggs hatch into larvae, which then penetrate the duodenal wall and enter the portal circulation. From there, they travel to the liver and eventually reach the lungs, where they undergo further transformation into larvae. The larvae of Ascaris migrate to the alveoli and ascend to the upper respiratory tract via secretions. They then go to the small intestine by being ingested along with sputum. In the small intestine, these parasites often grow into adults and often remain asymptomatic. However, in instances with a large number of worms, they may cause intestinal blockage or perforation peritonitis. It is important to consider these possibilities when diagnosing acute abdominal conditions, particularly in youngsters. This worm is capable of traversing the ampulla of Vater and causing hepatobiliary and pancreatic ascariasis. This condition is regarded as the second most common cause of biliary symptoms and pancreatitis in locations where the worm is prevalent [5,6].

Biliopancreatic ascariasis often manifests as biliary colic, acute cholangitis, acute cholecystitis, and acute pancreatitis. However, in 2.1% of instances, it may also lead to the formation of hepatic abscesses, which may include worms inside the abscess chamber. Ultrasonography is a rapid, secure, and noninvasive technique for diagnosing hepatic abscesses and ascariasis [7,8]. Ascaris-induced hepatic abscess may manifest with symptoms resembling an acute abdomen and should be considered as a potential diagnosis. We report the case of a child who presented with symptoms of acute abdomen and was diagnosed with acute intestinal obstruction caused by Ascaris infection.

## Case presentation

A 4.5-year-old boy from Khedi Sikarpur (Haridwar, India), presented to the pediatric department with complaints of abdominal pain for three days, not passing stool for three days, vomiting for one day, and abdominal distension for one day. There was a history of recurrent vague abdominal pain and pica for the past two months. The patient comes from a lower socioeconomic status family; his father is a laborer, and he has three siblings. There was no history of bleeding manifestations, decreased urine output, fever, passage of worms, or foreign body ingestion.

On examination, the patient exhibited pallor and a thin build, showing signs of malnutrition, with a weight of 10.2 kg, a height of 92 cm, and a head circumference of 48 cm. The weight-for-height, length-for-age, and weight-for-age were all below the 3rd percentile, while the head circumference-for-age was between the 3rd and 50th percentile. There was no edema or lymphadenopathy. His temperature was 98.8°F, heart rate was 98 beats per minute, and respiration was 21 breaths per minute. Other general examinations from head to toe were normal. On systemic examination, the abdomen was distended with an abdominal girth of 45.8 cm and generalized tenderness, especially in the right hypochondrium. There was rigidity in the central and mid abdomen. No organomegaly or shifting dullness was observed. On auscultation, decreased bowel sounds and minimal peristalsis were noted. Other systemic examinations were within normal limits.

**Figure 1:**
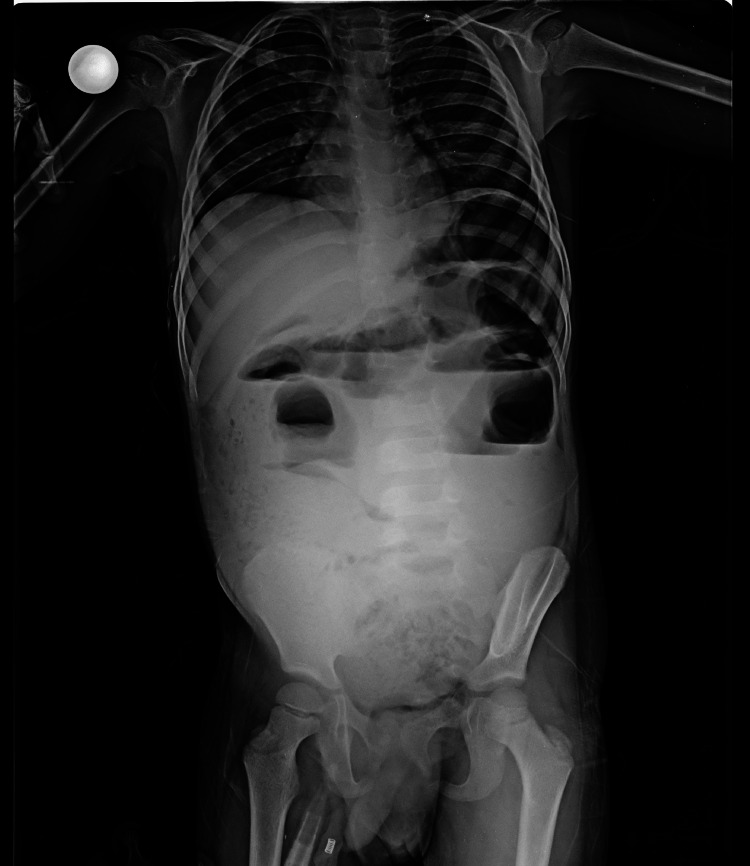
The X-ray of the abdomen showing multiple air-fluid levels

At the time of admission, lab investigations were performed. Hemoglobin was 9 mg/dL, and total leukocyte count (TLC) was increased to 17,000 per microliter, with a differential leukocyte count (DLC) of neutrophils 62%, lymphocytes 24%, and eosinophils 9%. Liver function tests (LFT), kidney function tests (KFT), blood urea nitrogen (BUN), and creatinine levels were within normal limits. An X-ray of the abdomen (anteroposterior (AP) view in erect posture) showed multiple air-fluid levels with no air under the diaphragm (Figure 1). An abdominal ultrasound (US) revealed parallel paired lines in the intestinal lumen, suggestive of worms. On the basis of history and examination findings, surgery opinion was taken.

**Figure 2:**
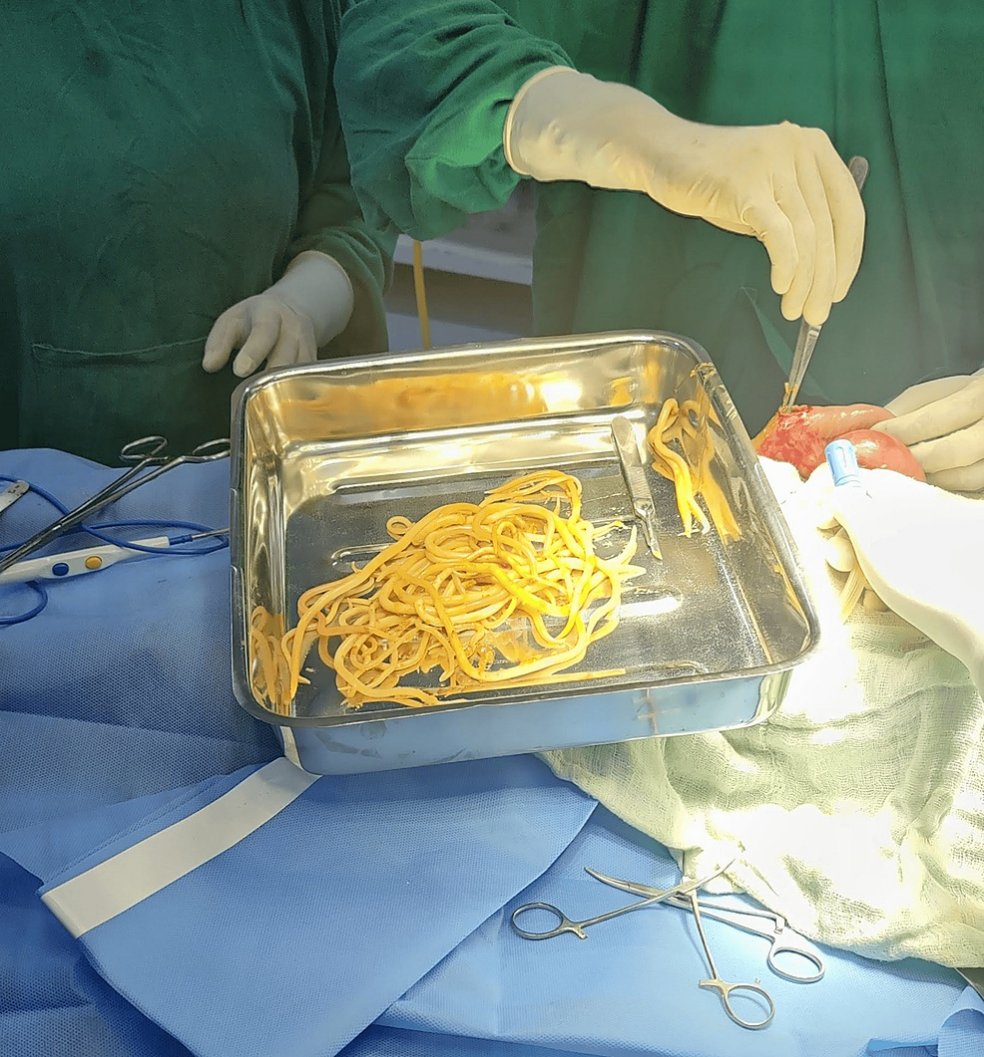
Multiple worms extracted from the small intestine

**Figure 3:**
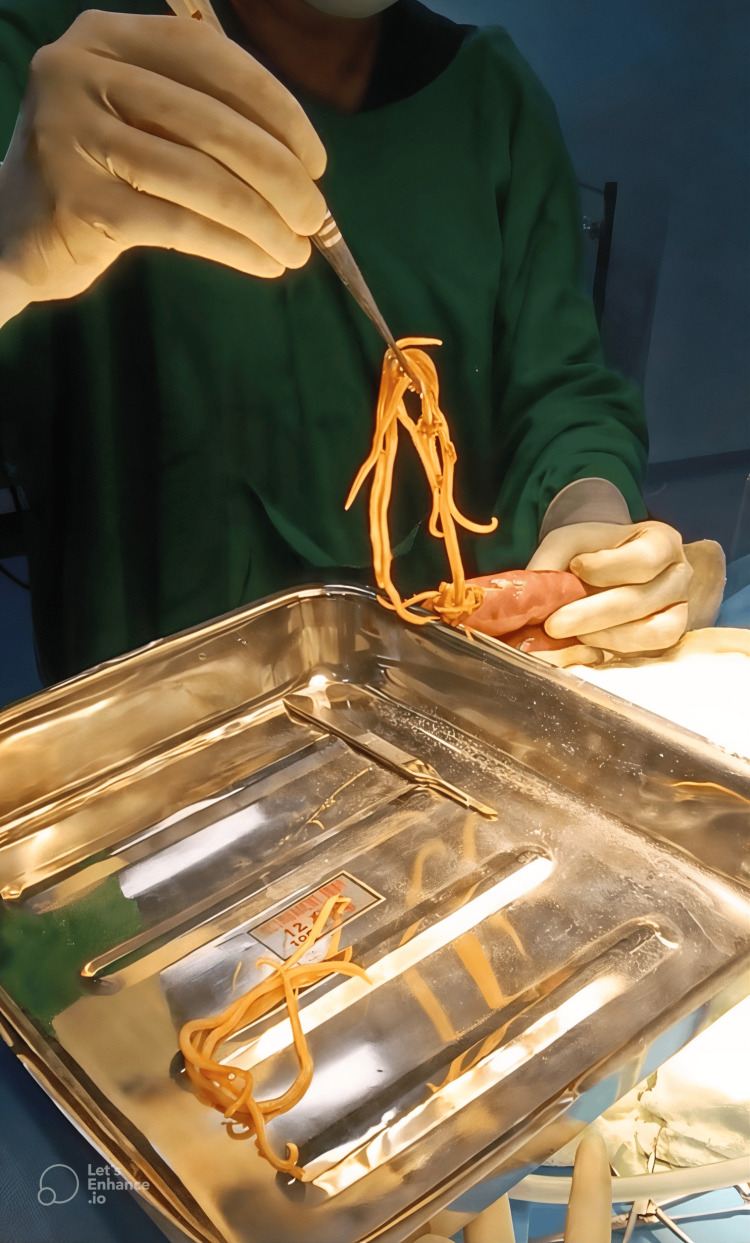
Worm extraction with the help of forceps

The patient was prepared for emergency exploratory laparotomy with IV antibiotics (ceftriaxone, gentamycin, and metronidazole) as prophylaxis, blood transfusion, and analgesics. During the exploratory laparotomy, multiple worms were found in the small intestine (Figure 2). Manual milking was performed to collect the worms, facilitating their extraction with forceps (Figure 3). No other intra-abdominal organ abnormalities were found.

**Figure 4:**
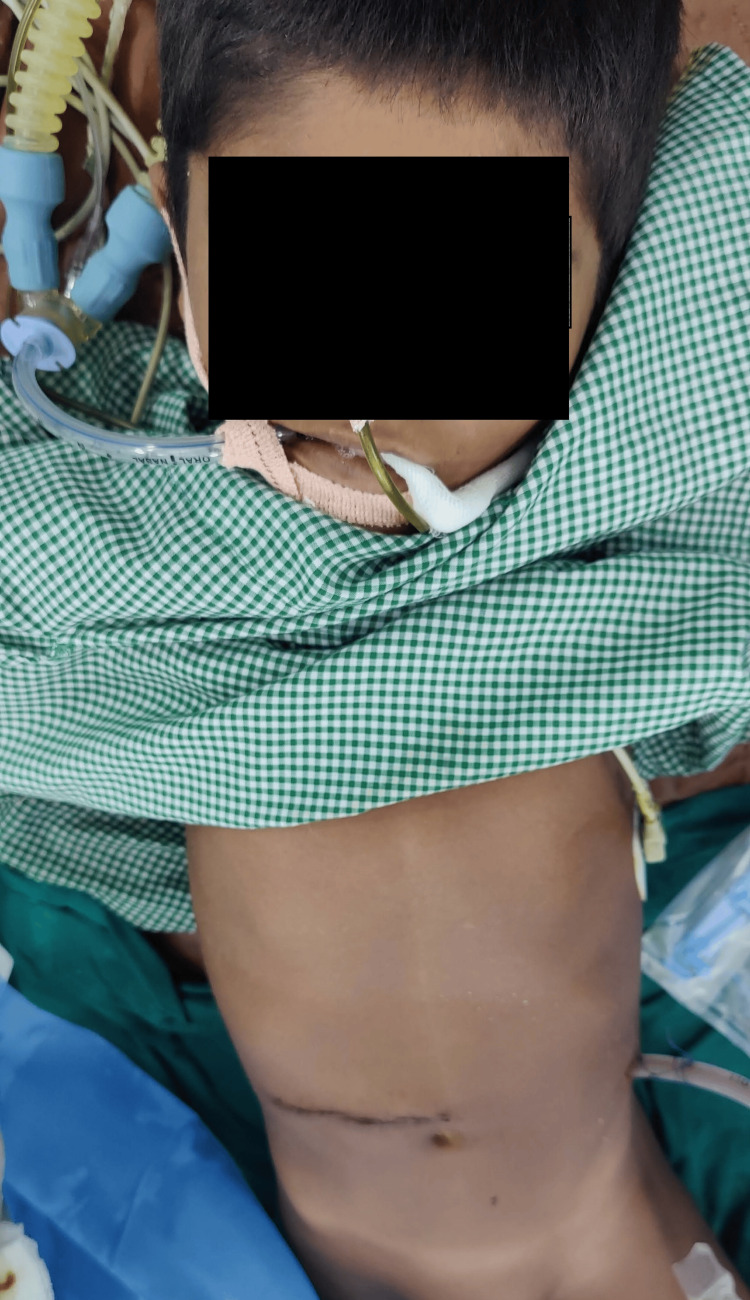
Postoperative image of the patient showing the abdominal suture line

Postoperative management involved IV fluids with Ringer's lactate, IV paracetamol, and IV antibiotics (Figure 4). Progressive oral sips were allowed eight hours after surgery, and semi-solid food was introduced on postoperative day three. The patient was hemodynamically stable and discharged on postoperative day seven. Deworming of the patient and his entire family was done with albendazole. Follow-up after one week showed a healed abdominal suture line with normal bowel functions. The dose of albendazole was repeated after two weeks.

## Discussion

Ascaris is the largest type of roundworm that may infect the human gut as an obligate parasite throughout its whole adult stage. This is mostly due to the regular practice of unsanitary disposal of human waste. In India, the incidence rate of Ascaris-induced intestinal blockage is 9.2 cases per 100,000 individuals. The primary means of *A. lumbricoides* infection is the consumption of embryonated eggs present in raw vegetables, water, or hands contaminated with dirt. The fertilized eggs emerge from the gut after hatching. The released larvae infiltrate the intestinal wall to reach the right side of the heart, the pulmonary circulation, and subsequently the alveoli. Upon expectoration by the host, the larvae are then ingested and undergo maturation inside the intestinal tract to reach adulthood. Its most frequent location is in the jejunum and ileum [9]. The adult roundworms deposit their eggs in the small intestine, which are subsequently excreted in the feces.

Ascaris typically resides without causing symptoms in the small intestine. However, in instances when there are a large number of worms, they may aggregate into a mass and lead to blockage or rupture of the small intestines. Worm bolus blockage is a significant factor in causing intestinal obstruction in children [10]. Roundworms may also travel via the ampulla of Vater to reach the biliary system and pancreas, resulting in biliary colic, acute cholangitis, acute cholecystitis, and pancreatitis. Occasionally, this worm may also generate hepatic abscesses and manifest as an acute abdomen.

Out of a total of 500 cases including hepatobiliary and pancreatic ascariasis, only four patients (0.8%) were found to have Ascaris-induced hepatic abscess [11]. In a separate study conducted in the same area, there were 510 instances of hepatic abscess. Out of these cases, 74 (14.5%) were determined to be caused by Ascaris. However, in only 11 cases (2.15%), the worm was detected within the abscess cavity [12]. The diagnosis of hepatic abscess caused by roundworms is easily determined when the worm is found within the abscess cavity. However, in cases when the worm is not present, the diagnosis may be suggested by examining the fluid from the abscess cavity.

Ultrasound is an effective method for diagnosing hepatic abscesses and ascariasis. It may readily identify roundworms as linear echogenic structures without shadowing. However, in some circumstances, other modalities like CT scan or MRI with magnetic resonance cholangiopancreatography (MRCP) may be necessary for accurate diagnosis. An endoscopic retrograde cholangiopancreatography (ERCP) is contraindicated for diagnosing hepatobiliary ascariasis. Its use should only be limited to therapeutic reasons [12-15].

Treatment for the patient with a hepatic abscess caused by Ascaris infection should include the use of antibiotics that are effective against a wide range of bacteria, medications that kill parasitic worms, and the evacuation of the abscess using a procedure called percutaneous aspiration or drainage. If possible, the worms should also be removed using endoscopic, percutaneous, or surgical procedures [12,13,15]. With proper hand hygiene practices and sanitation, we can avoid acquiring ascariasis infection. Education plays a vital role in achieving this. In children with ascariasis, a CT or US should be performed to rule out the possibility of a liver abscess, as such patients can have a liver abscess even without fever or right hypochondrium pain.

## Conclusions

Preschool children presenting with abrupt acute intestinal blockage should be considered with high suspicion of having a parasitic infection to avert severe and perhaps fatal consequences. Timely surgical intervention in individuals with acute intestinal blockage leads to a favorable prognosis. Conservative therapy may lead to spontaneous resolution of subacute intestinal blockage caused by* A. lumbricoides*. Incorporating information on ascariasis and its preventative measures into all health education programs is essential. This knowledge should be imparted to both school children and their mothers to mitigate the risk of infection. Timely identification, using the prevalence in the local area as a basis, may help avert severe surgical complications, illness, and death linked to intestinal blockage.
